# The utility of transposon mutagenesis for cancer studies in the era of genome editing

**DOI:** 10.1186/s13059-015-0794-y

**Published:** 2015-10-19

**Authors:** Gina M. DeNicola, Florian A. Karreth, David J. Adams, Chi C. Wong

**Affiliations:** Meyer Cancer Center, Weill Cornell Medical College, New York, NY 10021 USA; Experimental Cancer Genetics, Wellcome Trust Sanger Institute, Wellcome Trust Genome Campus, Hinxton, CB10 1HH UK; Department of Haematology, University of Cambridge, Hills Road, Cambridge, CB2 0XY UK

## Abstract

The use of transposons as insertional mutagens to identify cancer genes in mice has generated a wealth of information over the past decade. Here, we discuss recent major advances in transposon-mediated insertional mutagenesis screens and compare this technology with other screening strategies.

## Introduction

Genome sequencing has revealed a plethora of mutations in cancer, with some tumors carrying tens of thousands of somatic mutations [1]. Importantly, the relevance of these mutations is not always intrinsically clear and as a result must be inferred from the types of mutations observed, their frequency across tumor types, and their predicted effects on protein function. Insertional mutagenesis screens provide a functional readout to complement these sequencing studies, as genes identified by insertional mutagens are likely to represent both functionally important and evolutionarily conserved cancer genes. Insertional mutagenesis studies can also highlight cancer genes or common pathways that are disrupted at low frequency or by processes not immediately obvious from the genome sequence alone.

The first insertional mutagenesis efforts in mice were performed with the murine leukemia virus and the mouse mammary transforming virus to induce lymphoma and mammary tumors [2, 3], respectively, and led to the identification of numerous cancer pathways, including the WNT pathway [4]. However, these viruses were found to be of limited utility for mutagenesis in other tissue types owing to viral tropism and the fact that they only infect replicating cells [5]. Furthermore, as these retroviruses generate insertions that activate gene expression, they almost exclusively tag proto-oncogenes [5], restricting our ability to identify other types of cancer genes such as tumor suppressors.

**Fig. 1:**
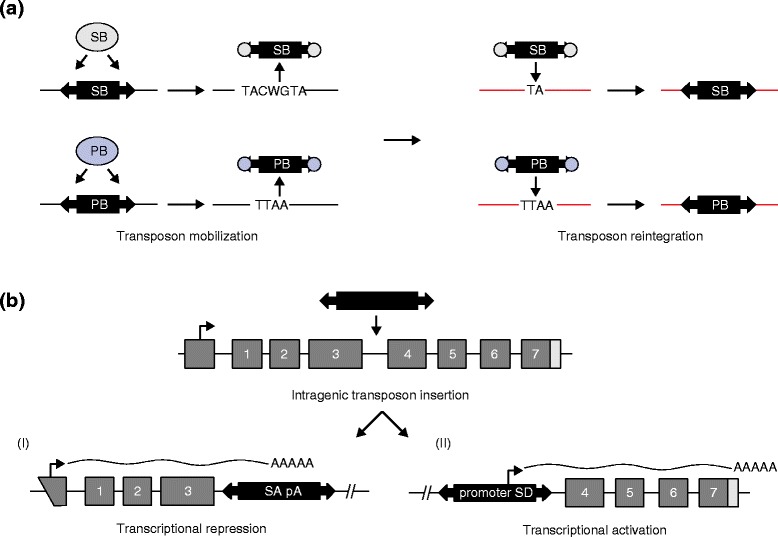
Transposons as insertional mutagens. **a** *Sleeping Beauty* (*SB*) and *piggyBac* (*PB*) (*black rectangles*) are mutagenic transposons that can be mobilized from donor loci (*left panel*) and reintegrated into other loci (*right panel*). Repeats in the transposon (*arrowheads*) are recognized by the *Sleeping Beauty* or *piggyBac* transposases (*ovals*), resulting in the transposon being excised from the genome. Reintegration of mobilized SB or PB transposons can occur at TA and TTAA sites, respectively, catalyzed by transposase activity. **b** Transposon insertion can promote or disrupt gene expression. In the example depicted in this panel, a transposon integrates between exons 3 and 4 (*numbered gray boxes*) of a gene. This can result in two possible outcomes: (I) the transposon disrupts gene function by hijacking transcription through the splice acceptor-polyadenylation signal (*SA-pA*) elements, leading to expression of a truncated transcript (exons 1–3); or (II) the transposon drives expression of the downstream gene sequences (exons 4–7) through the promoter-splice donor (*SD*) elements. Depending on the integration site, transposons can activate or abrogate expression of either the entire mRNA of a gene or only parts of it

For these reasons, DNA transposons were developed as insertional mutagens [6]. Transposons are mobile elements that move through the genome by a cut-and-paste process (DNA transposons), or through an RNA intermediate in a copy-and-paste mechanism (retrotransposons) [7]. Endogenous transposons are ubiquitous in vertebrate genomes, comprising approximately 45 % of DNA sequence [8], but are largely silent as a result of inactivating mutations acquired through evolution. The introduction of exogenous DNA transposons allows insertional mutagenesis in a wider spectrum of tissues than the ones that are accessible with retroviruses, and thus the generation of new mouse tumor models [9, 10]. The most commonly used transposon systems are the *Sleeping Beauty* (SB) and *piggyBac* (PB) systems [11]. A typical transposon used for in vivo insertional mutagenesis contains splice acceptors (SAs) followed by polyadenylation signals (pA) in both orientations, and a unidirectional promoter upstream of a splice donor (SD). A transposon can either disrupt gene function when it integrates into the body of a gene, thereby intercepting and curtailing transcription through the SA–pA elements, or it can activate expression when inserted upstream of a gene as the promoter–SD module drives expression of downstream sequences (Fig. 1). The pattern and orientation of transposon integration sites therefore often provide a clue as to whether the affected gene encodes a tumor suppressor or an oncogene.

Here, we discuss recent advances in cancer gene discovery using transposons and their role in the era of other mutagenesis tools such as clustered regularly interspaced short palindromic repeats/CRISPR-associated protein 9 (CRISPR/Cas9).

## Transposon-mediated insertional mutagenesis

In 2005, the groups of David Largaespada, Nancy Jenkins and Neal Copeland reported the use of the *Sleeping Beauty* transposon system as a tool for the identification of cancer-promoting genes in transgenic mice [12, 13]. Largaespada and colleagues performed whole-body transposon-mediated insertional mutagenesis (TMIM) with the first-generation T2/Onc transposon, accelerating tumorigenesis in mice null for the tumor suppressor *p19Arf* gene [12]. Using a more active transposon system (T2/Onc2), Dupuy and colleagues induced predominantly hematopoietic tumors following global mutagenesis in wild-type mice [13]. Following these landmark studies, a variety of transgenic mouse strains harboring different versions of transposons and transposases have been generated and utilized for candidate cancer gene discovery. By targeting SB transposase expression to tissues of interest, a variety of cancers have been generated by mutagenesis [13–20]. Additionally, several cancer types have been accelerated by TMIM in combination with sensitizing mutations [21–27, 29, 30] (Table 1). Collectively, many candidate cancer genes have been identified in the mouse that have subsequently been found to be relevant clinically and prognostically in human malignancies [20, 24] (Table 1). In a similar way, the PB transposon has been used for cancer gene discovery in the hematopoietic system and pancreas [31, 32].

**Table 1:** Capacity of TMIM screens to identify common human cancer genes in three cancer types^a^

Cancer type	Genes commonly mutated in human cancer	Recurrent genes identified by TMIM screens	Comments	Novel, functionally validated cancer genes identified in TMIM screens
Colorectal	*APC* [102]	Yes [19, 22, 23, 51]		*CNOT1*, *PDE4DIP*, *PDCD6IP*, *ATF2*, *SFI1* [22]
	*TP53* [102]	Yes (low frequency) [23, 51]	*Tp53* has been rarely targeted in any TMIM screen, although upstream regulators such as *Cdkn2a* are frequently targeted in TMIM screens	
				*ANKRD11*, *CSNK2A1*, *MKL2*, *MYO9A*, *RNF43*, *SIN3A*, *Zfp292* [51]
	*KRAS* [102]	Yes (low frequency) [23, 51]	Insertions in *Kras* have been detected at low frequency in *Apc*-deficient backgrounds [23], perhaps reflecting the inability of transposons to cause point mutations in target genes through insertion alone. However, upstream and downstream genes within the Kras signaling pathway are targeted in TMIM screens, leading to pathway activation	
	*PIK3CA* [102]	No	Other phosphoinositide 3-kinase pathway genes have been targeted, for example *Pik3r1* and *Pten*	
	*FBXW7* [102]	Yes [23, 51]		
	*SMAD4* [102]	Yes [23, 51]		
	*CTNNB1* [102]	Yes [23, 51]		
	*NRAS* [102]	No	Recurrent insertions in *Nras* have not been found, probably owing to the same reasons applicable to *Kras*	
	*TCF7L2* [102]	Yes [23, 51]		
	*FAM123B* [102]	No	Other components of Wnt–β-catenin pathway are targeted in tumors, for example *Apc* and *Ctnnb1*.	
Melanoma	*BRAF* [103, 104]	Yes [49]	Identified as potential mediator of BRAF inhibitor resistance	*ERAS* [49]
	*NRAS* [103, 104]	No		
	*TP53* [103, 104]	No		*CEP350* [38]
	*PTEN* [103, 104]	Yes [38, 49]		*MAGI2*, *PTPRO*, *Map3k1* [57]
	*CDKN2A* [103, 104]	Yes [38, 49, 57]		
	*NF1* [103, 104]	Yes [38]	Melanoma TMIM screens were performed in a *Braf*-mutant background. *NF1* mutations are typically mutually exclusive from *BRAF* mutations in human melanoma, potentially accounting for the lack of *Nf1* insertions in some screens	
	*RAC1* [103, 104]	No	Other genes operating in Rho GTPase pathways have been targeted	
	*MAP2K1* [103, 104]	No	Other MAP kinase pathway genes have been targeted in melanoma TMIM screens	
	*ARID2* [103, 104]	No		
	*PPP6C* [103, 104]	Yes [38, 49]	Identified as potential mediator of BRAF inhibitor resistance	
Pancreatic	*KRAS* [105, 106]	Yes (low frequency) [24]	Pancreatic cancer TMIM screens were performed in a *Kras* ^*G12D*^ background	*USP9X* [24]
	*TP53* [105, 106]	No	Recurrent insertions in *Tp53* were not observed despite the high prevalence of *TP53* alterations in human pancreatic cancer. *Cdkn2a* was a recurrent CIS-associated gene, and *Usp7*, a p53-regulatory deubiquitinase, was targeted at low frequency in one study [24]	*Foxp1*, *Foxp2*, *Bcl6*, *Fign* [32]
	*CDKN2A* [105, 106]	Yes [24, 32]		
	*SMAD4* [105, 106]	Yes [24, 25]		
	*PREX2* [106]	No		
	*TGFBR2* [105, 106]	Yes [24, 32]		
	*RNF43* [106]	Yes [24, 25]		
	*KDM6A* [106]	Yes [24, 25, 32]		
	*ARID1A* [105, 106]	Yes [24, 32]		
	*MLL3* [105]	Yes [24, 25]		

^a^Shown are details of TMIM screens for identifying common human cancer genes in three types of cancer for which more than one screen has been performed. *TMIM* transposon-mediated insertional mutagenesis

## TMIM — technical considerations

**Fig. 2:**
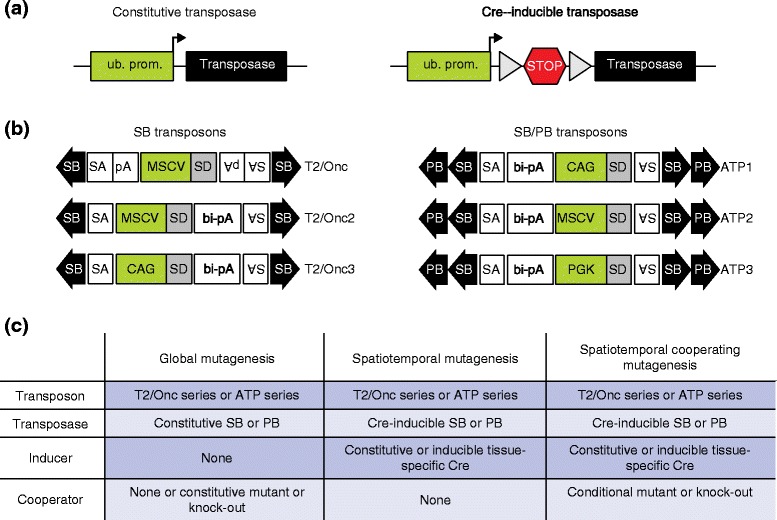
Tools for transposon-mediated mutagenesis. **a** Transposase expression can be either ubiquitous (*ub. prom.*) or directed to a particular cell or tissue type by using Cre-inducible alleles of the transposase enzyme. In the latter case, a *lox*P-site-flanked transcriptional stop element (*gray triangles* and *STOP sign*, respectively) prevents transcription of the gene encoding transposase. Upon Cre-mediated excision of the *lox*P-STOP-*lox*P element, the transposase is expressed in Cre-positive cells. **b** A variety of transposons have been developed for mutagenesis. SB transposons have been developed that carry either a murine stem cell virus (*MSCV*) promoter (T2/Onc and T2/Onc2) or the chicken β-actin/CMV enhancer (*CAG*) promoter (T2/Onc3). To facilitate gene activation, transposons carrying these promoters also contain splice donor (*SD*) elements, and, for gene disruption, splice acceptor (*SA*) and polyadenylation (*pA*) elements (*bi-pA* bi-directional polyadenylation signal). Versatile SB/PB transposons containing terminal repeats recognized by SB and PB transposases (*arrowheads*) have also been developed and carry either CAG, MSCV or mouse phosphoglycerate kinase 1 (*PGK*) promoters (ATP1, ATP2 and ATP3 transposons, respectively). **c** Using combinations of the aforementioned alleles tabulated here, global or spatiotemporal mutagenesis with co-operating mutations can be performed

Various mouse strains have been generated that express SB or PB transposase in a ubiquitous or conditional manner. With these strains, transposon mobilization can be induced either in the whole animal or in a tissue- or temporal-restricted manner by using an appropriate Cre recombinase allele (Fig. 2). The transposon mice are transgenic strains containing transposon concatemers on a single chromosome. As a consequence, many insertion sites are found locally, and the tendency for local integrations is reported as being higher with SB compared with PB [33]. The number of transposons in the concatemer is also a consideration. Global mobilization of greater than 20–30 transposon copies during embryonic development correlated with increased embryonic lethality [13, 15, 31]. Additionally, increasing transposon numbers amplifies the potential for passenger integrations, which do not contribute to the observed phenotype.

The promoter within the transposon can display tissue-specific activity and thereby influence the phenotype of whole-body insertional mutagenesis screens or the insertion sites that are positively selected for in organ-specific screens. Indeed, the first transposon mouse strains (T2/Onc, T2/Onc2) utilized the murine stem cell virus (MSCV) promoter, which displays a propensity for the development of hematopoietic tumors. However, replacing the MSCV promoter with the chicken β-actin/CMV enhancer (CAG) promoter or the phosphoglycerate kinase 1 (PGK) promoter significantly increased the incidence of solid tumors in both the SB and PB system [14, 31]. Thus, the modularity of transposons and the ability to modify elements such as the promoters they carry can be used to influence the tumor type and incidence.

**Fig. 3:**
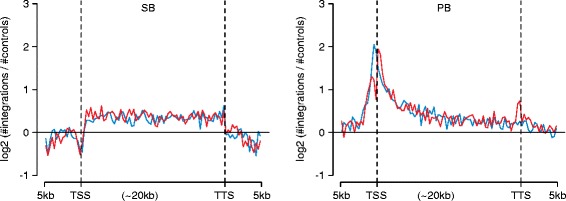
Integration biases of SB and PB transposons. The distribution of transposon insertions across genes from 5 kb upstream of the transcription start site (*TSS*) to 5 kb downstream of the transcription termination site (*TTS*). *Red*, transposon insertions in the sense orientation relative to the gene; *blue*, insertions in antisense direction. Reproduced from [34]

An important technical consideration in transposon screens is integration bias. SB has been reported to demonstrate a bias towards integration into DNA sequences containing TA nucleotides and appears to preferentially integrate into gene bodies but not into transcriptional start sites (TSSs) [34] (Fig. 3). Conversely, PB, which predominantly integrates into TTAA sequences, displays a preference towards integration into TSSs over gene bodies (Fig. 3). As a consequence, oncogenes are more likely to be identified using PB, whereas transposon integration in tumor suppressors is primarily seen when the SB system is used, but this again is influenced by the promoter elements used in the transposon. Allan Bradley’s group recently reported the development of a conditional PB transposase mouse allele [32], which can direct cell- or tissue-specific expression of PB, and hence directs mutagenesis to a specific cellular compartment. The development of this strain allowed the direct comparison of screening data generated in a mouse model of *Kras*^*G12D*^*-*driven pancreatic cancer, where a prior screen with the SB transposon system had been performed [24]. The PB screen identified candidate drivers that had also been identified by the pancreatic SB screen as well as novel candidate pancreatic cancer genes, and thus exemplified the complementarity of the SB and PB approaches as in vivo insertional mutagens for cancer gene discovery.

Another consideration that investigators should be mindful of when performing insertional mutagenesis screens is the damage done to the genome by the process of transposition itself as transposons are mobilized from chromosomal integration sites. Excision of PB transposons generally results in no or limited damage to the genome; by contrast, the mobilization of SB transposons leaves behind a two-to-five nucleotide footprint [35]. SB transposon footprints can thus result in frameshift mutations, splicing alterations or promoter disruptions, which in turn could promote tumorigenesis. The mobilization of transposons in *cis* could also result in chromosomal rearrangements such as deletions or copy-number-neutral changes [36]. Fortunately, these passenger effects appear to be limited [36–38], and thus tumor promotion in transposon screens appears to be largely driven by transposon insertion events, but this factor is nevertheless of consideration in the analysis of tumors collected during screening.

## TMIM — statistical considerations

Although tumor evolution selects for mutagenic insertions that drive tumorigenesis, each tumor cell will harbor multiple additional inconsequential passenger insertions, as repeated rounds of transposon mobilization and reintegration will result in thousands of integration sites in a polyclonal tumor. Cancer drivers cannot be identified solely by sequencing all of the insertion sites in a given tumor — this merely gives a snapshot of insertion sites at a point in time. Thus, statistical approaches are necessary to reveal regions of the genome that are enriched with insertions more than expected by chance — so-called common insertion sites (CISs). By mapping CISs onto a reference genome, CIS-associated genes can be identified as potential cancer drivers.

A number of statistical approaches have been used to identify CIS-associated genes from transposon screens. Early studies deployed Monte Carlo-based methods and Poisson distributions [39, 40] to define those genomic locations enriched with insertion sites. More recently, Gaussian Kernel Convolution (GKC) approaches [41], gene-centric common insertion site (gCIS) analysis [42] and refined versions of the Poisson approach have been developed [43]. Essentially, all these methods provide a measure of the degree to which insertion sites are enriched at a given locus relative to either a pre-computed background distribution or an insertion dataset derived from tissues in which transposons have been mobilized for a short period of days or weeks, before clonal selection could be operative. The concordance between methods ranges between 60 and 80 %, and thus most investigators use multiple algorithms to identify CISs [23]. Methods such as GKC [41] adjust the significance statistic for a locus (CIS) relative to the frequency of the transposon target site (TA for SB, and TTAA for PB) that can account for some local biases in transposon integration. Both the type and stringency of the CIS-calling methods used to identify insertions affect the classification of co-occurring or mutually exclusive CISs. Reinders and colleagues have developed a two-dimensional GKC method to identify co-operating mutations from virally induced mutagenesis data, a method that has also been applied to TMIM screens [44]. In addition, the Poisson regression insertion model (PRIM) [45] has been used to identify co-occurring gene pairs, and the TAPDANCE algorithm can generate the association of independent CISs by using a Fisher’s exact test [43].

## Limitations of TMIM

TMIM is a powerful tool for in vivo cancer gene discovery, but, as with every technology, there are several limitations. We summarize these limitations here and also allude to them throughout the text. The primary limitation is the inability of the transposons to interrogate the genome in a completely unbiased fashion. Transposons do not integrate into and affect all genes with similar probability owing to factors such as promoter selection within the transposon [31], integration-site preferences [34], local transposon hopping [33], gene size (larger genes are more likely to be affected by transposon integrations) and the relative superior ease of isolating tumor suppressors as the precise transposon integration site and orientation with respect to the target gene are less crucial factors for tumor suppressors compared with those of oncogenes.

Another limitation is that TMIM cannot recapitulate the complete spectrum of mutations that are commonly found in human cancer, such as point mutations. Elevated expression and mutations may not result in identical biological outcomes, and thus transposon-mediated overexpression of proto-oncogenes does not always mimic the effects of somatic, gain-of-function point mutations [46]. Similarly, mutations in tumor suppressors can result in dominant-negative effects that are not recapitulated by transposon-insertion-mediated loss of expression [47]. The insertion spectrum recovered by TMIM screens can also be affected by the sensitizing genetic backgrounds that activate pro-tumorigenic pathways — for example, oncogenic mutants of B-Raf or Kras [24, 32, 38, 48, 49], such that genes that activate the same pathway as the sensitizing mutation are unlikely to be identified in these particular backgrounds. Finally, transposon insertions are unable to recapitulate reciprocal translocations such as *BCR*–*ABL* and other genomic alterations that commonly occur in cancer.

There are also technical and resource limitations to TMIM approaches. For example, investigators might wish to perform drop-out screens designed to identify genes that are detrimental to cells when mutated. Such screens are not feasible with TMIM as such cells are lost during the screening process. Moreover, the generation of mouse cohorts is both time-consuming and costly for in vivo TMIM screens as compound mutant mice carrying three or four transgenic alleles are typically required. Finally, candidate cancer genes identified through TMIM screens in the mouse might not necessarily have equal relevance in human cancer — follow-up validation studies must therefore be performed. Investigators should consider all these limitations when designing transposon screens.

## Transposon mutagenesis — beyond the basic screen

Over the past decade, numerous TMIM studies have identified known and novel cancer genes that either promote tumor initiation or co-operate with cancer-sensitizing mutations to drive tumor progression. Recently, novel and elegant ways of employing transposon mutagenesis to query specific cancer processes have been devised. In this section, we summarize recent developments in the TMIM field.

### Investigating tumor progression and evolution

TMIM screens have been performed in mice harboring various initiating mutations found in human cancer. Such screens identify drivers of tumor progression and, importantly, might be influenced by the sensitizing mutation. For example, Alexander and colleagues performed TMIM in the hematopoietic system, which resulted in multiple leukemias [50]. A *Jak2*^*V617F*^-mutant background skewed the disease towards erythroleukemia, and insertions in the ETS transcription factor genes *Erg* and *Ets1* were identified as the most common events. Conversely, when using an activated *ERG* allele (*TLS-ERG*) as the sensitizing mutation, the authors identified frequent activating insertions in *Jak2*, thus validating the co-operation between *Jak2* and *Erg* [50].

**Fig. 4:**
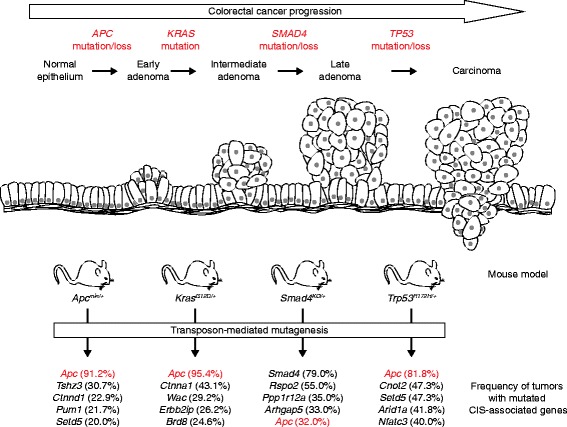
Use of transposon-mediated insertional mutagenesis (TMIM) screening to identify mutations that co-operate with specific genetic lesions associated with different stages of colorectal cancer development. The top panels illustrate a model of colorectal cancer initiation and progression [101], along with genetic alterations associated with these stages. TMIM screens using mouse models carrying mutations in corresponding genes have revealed that *Apc* was the predominant gene inactivated in tumors from all sensitizing genotypes apart from *Smad4* ^*KO/+*^ cases, where inactivation of the remaining wild-type *Smad4* gene is the most frequent insertional event

In an elegant study, TMIM was utilized to delineate evolutionary events during the progression of colorectal cancer (CRC) [51]. Jenkins, Copeland and colleagues crossed the SB system into different sensitizing backgrounds that carry mutations in genes that act at different stages of CRC: *Apc*^*min*^, *Kras*^*G12D*^, *Smad4*^*+/−*^ or *Tp53*^*R172H*^ (Fig. 4) [51]. Intriguingly, this approach revealed that functional loss of the wild-type *Apc* allele was the most crucial event for tumor progression in *Apc*^*min*^, *Kras*^*G12D*^ and *Tp53*^*R172H*^ tumors, but not in tumors that were initiated by heterozygous loss of *Smad4*. Instead, those tumors displayed frequent insertions in the wild-type *Smad4* allele along with mutually exclusive insertions in *Rspo1* and *Rspo2* that promoted overexpression of these R-spondins, which are known enhancers of Wnt signaling. In addition, 111 candidate cancer genes were identified that were independent of the initiating mutation.

These studies illustrate how sensitizing mutations can co-operate with transposon-associated lesions and how different pre-existing mutations can sometimes influence the trajectory of subsequent mutation acquisition during tumor development. In the case of human CRC, loss of *APC* is thought to be the initiating event, whereas mutations in *KRAS*, *TP53* or *SMAD4* occur later during tumor progression. Indeed, transposon-insertion-mediated loss of *Apc* appeared to be a prerequisite for colon tumorigenesis in the *Apc*^*min*^, *Kras*^*G12D*^ and *Tp53*^*R172H*^ backgrounds, whereas insertions in *Kras* and *Tp53* are rare in *Apc*-loss-driven CRC [51] (Table 1; Fig. 4). This finding further supports the notion of *APC* being the gatekeeper of CRC. Conversely, leukemogenesis is initiated by either mutant *Jak2* or *Erg* and progresses upon transposon insertions in the other gene, suggesting that the temporal sequence of mutation might be irrelevant [50]. Taken together, TMIM is a valuable tool to delineate tumor progression, and future studies that unravel the genetic dependencies of co-operating mutations on different initiating mutations in other cancer types will shed further light on the genetics of tumor progression and might be useful for devising treatment strategies.

Determining the evolutionary history of mutations within tumors can inform our understanding of the mutational forces that shape cancer development. To assess tumor clonality in a more quantitative fashion, new methods to estimate the frequency of transposon insertions in tumors have been devised. Historical methods to retrieve insertion sites have been based primarily on PCR amplification of restriction-endonuclease-digested, adaptor-ligated tumor DNA, followed by high-throughput sequencing. However, sequence coverage cannot be used to infer tumor clonality accurately owing to PCR biases as a result of the variable distribution of restriction enzyme sites in the genome. An alternative approach, called shear-splink, was developed by Jonkers and colleagues that fragments DNA by acoustic shearing, mitigating this bias [52]. In addition, as DNA is fragmented at random, each fragment harbors a potentially unique stretch of DNA that can serve as a molecular barcode. Quantification of these barcodes permits estimation of transposon clonality within a heterogeneous sample. Rad and colleagues used a similar approach, termed quantitative insertion site sequencing (QIseq), to illustrate the marked genetic complexity of pancreatic tumors [32]. Although these approaches can estimate transposon clonality, they cannot distinguish between transposon heterogeneity arising during tumor evolution in a monoclonal sample and multiple distinct insertions in a polyclonal tumor population.

### Identifying genes involved in metastasis

In addition to identifying genes involved in tumor initiation and progression, TMIM has been performed to discover genes that promote tumor dissemination. Largaespada and colleagues expressed the SB system in p53-deficient mouse osteoblasts and identified candidate genes involved in metastasis by comparing transposon insertions from osteosarcoma metastases with those found in primary tumors [53]. Approximately one-third of CIS-associated genes found in metastases were evident in primary tumors. Furthermore, from this analysis, five candidate oncogenes and 38 tumor suppressors were identified, including nine genes that have been implicated previously in cancer metastasis. To study further the evolutionary relationships between metastases and parental ancestors, the authors conducted parsimony analysis of tumors using transposon integration sites as molecular footprints. Osteosarcoma metastases were found to be highly clonal but appeared to show different patterns of evolution from the primary tumor.

Taylor and colleagues performed a TMIM screen aimed at identifying genes affecting dissemination of medulloblastoma in *Ptch1*^*+/−*^ heterozygous null or mutant *Tp53* mouse backgrounds [54]. Interestingly, the authors found that both transposon-driven mouse and human metastatic medulloblastoma are clonal but divergent from the primary tumor, suggesting that only a rare subclone in the primary tumor is able to metastasize. Four of the identified candidate genes were validated as drivers of medulloblastoma dissemination by retroviral delivery of these candidates to the cerebellum in combination with overexpression of the Ptch1 ligand sonic hedgehog (Shh) [55]. These studies demonstrated the utility of TMIM screens to discover drivers of metastatic spread, and further studies will identify candidate metastasis genes in certain genetic backgrounds and tumor types. Some mouse cancer models might not be suitable for identification of metastasis genes by TMIM because the mice have to be sacrificed before the formation of macroscopic metastases owing to the primary tumor size. However, surgical removal of the primary tumor to allow more time for metastasis growth or transplantation of primary tumor cells into syngeneic wild-type mice could circumvent this issue. Nonetheless, these reports illustrate how TMIM can be employed to query the clonal relationship of a primary tumor and its metastases, complementing the use of transposons to identify genes involved in tumor progression.

### Identifying alterations in cancer pathways

Apart from identifying genes promoting tumor progression, TMIM screens have been used to define the most prominent signaling pathways deregulated in tumors. Using the TAPDANCE tool, Largaespada and colleagues performed a pathway-centric analysis of alterations in *Tp53*-mutant, EGFR-driven peripheral nerve sheath tumors to identify roles for the phosphoinositide 3-kinase (PI3K)-AKT-mTOR, mitogen-activated protein kinase (MAPK) and Wnt/β-catenin pathways in the development of this tumor type [56]. Novel pathways have also been revealed in melanoma driven by oncogenic *B-Raf*^*V600E*^. Xu and colleagues identified a network involving Magi2 with a PB screen at low transposon copy number and also found insertions in *Map3k1* and *Map3k2* that resulted in ERK activation [57]. However, these insertions occurred in melanomas that had not recombined the conditional oncogenic *B-Raf*^*V600E*^ allele. Although not examined, this suggests that aberrant MAP3K1/MAP3K2 activation could represent another means to activate the MAPK pathway in human melanoma besides the common *BRAF* and *NRAS* mutations. The melanoma SB screen performed by Jenkins, Copeland and colleagues identified numerous candidate cancer genes, and pathway analysis found significant enrichment of CIS-associated genes in many cancer-related signaling pathways, including Wnt/β-catenin, TGF-β, PI3K and MAPK signaling, as well as in many biological processes [38]. Recently, it was shown that, by integrating SB TMIM in mice and mutation analysis of human cancer genomes, loss of function of the transcription factor CUX1 drives myeloid malignancy and other cancer types [20]. It was demonstrated that CUX1 antagonizes the PI3K–AKT signaling pathway by regulating transcription of the PI3K inhibitor PIK3IP1. Finally, a SB medulloblastoma screen in *Ptch1*^*+/−*^mice identified candidate cancer genes and associated protein networks capable of distinguishing the molecular subgroups of human medulloblastoma, demonstrating the power of transposon screens to recapitulate the genetic changes in human cancer [58].

These studies suggest that pathway and network analyses can provide insight into mechanisms of human disease and might predict survival and treatment outcomes. Thus, TMIM is a powerful approach to unravel the functional association of altered signaling pathways or cell-biological processes with cancer development. Conventional sequencing efforts can fail to identify such associations because the mutation rate of individual genes regulating these pathways or processes is not above the background mutation rate. Moreover, although TMIM cannot recapitulate activating mutations of proto-oncogenes, pathway analyses of TMIM datasets can reveal the crucial functions downstream of oncogenes that are commonly mutated in human cancer.

### Identification of novel mechanisms of gene deregulation

In cancer cells, loss of mRNA and protein expression can occur without any obvious genetic alteration in corresponding protein-coding regions. Notably, recent TMIM studies have identified novel non-coding regulatory regions and other mechanisms of gene deregulation that promote tumorigenesis. For example, a PB screen identified recurrent transposon insertions in a 200-kb noncoding region (Ncruc) upstream of the *Cdkn2a* gene [32], which encodes the tumor suppressors p16Ink4a and p19Arf and is frequently inactivated by prototypic gene-body insertions in both SB and PB pancreatic cancer screens [24, 32]. Transposon insertions in or genomic loss of the Ncruc region were associated with reduced expression levels of *Cdkn2a* in *cis*, demonstrating the power of PB insertional mutagenesis screens to identify non-coding DNA regions or genes with crucial roles in tumorigenesis.

Although target-site preferences suggest that PB-based TMIM screens might be more useful to identify regulatory elements compared with SB transposons (Fig. 3), SB-mediated screens have also been fruitful in identifying atypical mechanisms of gene deregulation in cancer. For example, Dupuy and colleagues performed a SB-mediated hepatocellular carcinoma (HCC) screen and found frequent insertions in the complex imprinted *Dlk1-Dio3* locus. A domesticated retrotransposon, *Rtl1*, located in this locus was shown to be overexpressed in all tumors with *Dlk1-Dio3* insertions [59]. Furthermore, ectopic overexpression of *Rtl1* in mouse livers induced HCC, validating *Rtl1* as a novel cancer driver. Examination of human liver tissue showed that *Rtl1* is transcriptionally inactive in normal liver but can be reactivated in human HCC, supporting a role for *Rtl1* in human HCC development.

In a SB-mediated TMIM screen aimed at identifying genes that co-operate with oncogenic *B-Raf* in melanoma development, a significant enrichment of genes was discovered among the CISs that encode mRNAs with the ability to regulate the expression of the tumor suppressor *Pten* [48]. These so-called competitive endogenous RNAs control Pten levels as microRNA decoys, in a protein-coding-independent fashion. While these CIS-associated genes are classical protein-coding genes, our analysis highlighted a non-coding function of their mRNAs. Only 2 % of the mammalian genome encodes protein-coding genes; however, the non-coding portion of the genome, both transcribed (e.g., microRNAs, long non-coding RNAs) and non-transcribed (e.g., enhancers), plays crucial roles in physiology and pathology. TMIM screens have barely scratched the surface of the non-coding space, and re-analyzing existing SB and PB mutagenesis data might reveal additional non-coding insertion hotspots.

### Identifying mechanisms of resistance to therapy

TMIM has been useful in identifying genes that mediate therapeutic drug resistance both in vitro and in vivo. Schmidt and colleagues conducted a PB screen in four different human cell lines derived from neuroblastoma, breast and cervical cancer to identify genes whose overexpression mediates resistance to paclitaxel [60]. Interestingly, while the authors identified multiple CISs in the four cell lines, the only CIS that was common to all four cell lines was the *ABCB1* gene [60], which encodes an ABC-transporter associated with multi-drug resistance [61]. This suggests the existence of both cancer-type-specific and common mechanisms of drug resistance. In addition, Xu and colleagues performed a PB screen in melanoma cells and identified BRAF and CRAF as mediators of resistance to the BRAF inhibitor vemurafenib [62], recapitulating previous observations in human melanoma patients and cell lines treated with vemurafenib [63–65].

In diploid cells, biallelic inactivating transposon insertions that completely abrogate gene expression are rare compared with monoallelic events, thus hampering the identification of genes that promote drug resistance only upon complete loss of expression. To tackle this issue, Ashworth and colleagues [66] took advantage of a haploid mouse embryonic stem (ES) cell system to screen for mediators of olaparib toxicity, in which inactivating transposon insertion can result in complete loss of gene expression. The authors identified the poly [ADP-ribose] polymerase 1 gene *Parp1* as a mediator of olaparib toxicity, and their results suggested that loss of Parp1 could result in olaparib resistance in patients [66]. In another mouse ES cell screen, Jonkers and colleagues identified loss of the gene *53bp1* as a mediator of survival and DNA-damage responses in *Brca1*-null cells [67]. Reduced 53BP1 expression was associated with basal-like, triple-negative, and *BRCA1/2*-mutant breast cancer in humans, suggesting that downregulation of 53BP1 might be an important survival factor in such tumors, particularly during chemotherapy-induced DNA damage. These studies demonstrate the utility of TMIM to identify mediators of resistance in human cancer cell lines as well as ES cells.

Drug resistance in patients develops in the context of a supporting microenvironment and, thus, in vitro approaches might be limited in their ability to identify resistance genes. To avoid this shortcoming of in vitro drug-resistance screens, a SB screen in a *B-Raf*^*V600*E^-driven mouse model of melanoma was performed. This identified transposon insertion sites in treatment-naïve tumors as well as melanomas treated with the vemurafenib progenitor compound PLX4720 [49]. Insertions in several known mediators of resistance were enriched in the PLX4720-treated tumors, validating this approach for resistance gene discovery. An ERAS-AKT-BAD signaling axis was validated as a mediator of drug resistance, which mimics the paracrine mechanism of stromal hepatocyte growth factor-mediated resistance [68, 69]. Curiously, many of the genes that have been previously identified in cell lines as promoters of resistance through reactivation of MAPK signaling were not identified in this in vivo study. A possible explanation is that such mutations are preexisting in patients only in a minor tumor subclone that no longer relies on oncogenic BRAF signaling. Conversely, transposon mobilization was induced concomitantly with the initiating B-Raf mutation in the resistance TMIM. In these tumor cells, transposon insertions that would otherwise result in MAPK activation might be negatively selected owing to functional redundancy with oncogenic B-Raf. Thus, additional insight might be gained from studies in which transposon mobilization is induced at the time of drug treatment.

### Novel approaches of employing transposon mutagenesis

In vivo transposon mutagenesis requires up to four transgenic alleles to accelerate tumorigenesis in a tissue-specific manner in a sensitizing background. Generating and maintaining compound mutant mouse strains is time consuming and costly, prompting alternative ways of utilizing the transposon systems. Molyneux and colleagues transduced immortalized primary human bone mesenchymal cells with SB and a lentivirus harboring the elements of a SB transposon, and, when injected into mice, the transplanted cells produced myxofibrosarcomas [70]. For human candidate cancer gene discovery, both the insertions of the parental lentivirus as well as the remobilized transposons were mapped. In another study, neural stem cells were derived from transgenic mice harboring the SB system and a Nestin-Cre allele [71]. Following in vitro differentiation, the neural stem cells were immortalized through SB mutagenesis and the resulting immortalized astroglial-like cells were injected into SCID mice to identify genes that drive glioblastoma formation. CIS mapping of immortalized cell lines and tumors identified partially overlapping CISs, suggesting differential roles of the identified genes during immortalization and tumorigenesis. In vitro delivery of the transposon system components followed by orthotopic or subcutaneous transplantation thus represents another means for in vivo selection and identification of candidate cancer genes.

The SB transposon system has also been used as a reverse-genetics tool to validate candidate cancer genes. Futreal and colleagues created transposons with both SB and PB terminal repeats that also harbored IRES-cDNA cassettes [72], such that the cDNA cargo was expressed only when transposon insertion occurred in transcribed genes. Using these transposons, the authors tested kinases with point mutations encoding putative gain-of-function oncogenic alleles. Mice were generated carrying multiple transposons with different cDNA cargos and crossed to SB transgenic mice, leading to tumorigenesis by in vivo selection of the kinase mutants with the highest oncogenic potential in somatic cells. This report elegantly displays how the transposon system can be utilized to discern the relative oncogenic properties of several candidate genes simultaneously in all or selected organs.

To extend the utility of TMIM to another model system, transgenic rats carrying the components of the SB or PB system have been created [73]. The transposons carried both SB and PB terminal repeats as well as a tyrosinase expression cassette, permitting coat-color-based phenotyping for transposon zygosity and genomic position effects on tyrosinase expression in albino rat backgrounds. In the future, it will be interesting to determine the overlap in cancer genes identified by TMIM screens in mouse and rat and their relevance to human cancer.

## Comparison with other technologies

Other methods of forward-genetic screens for the promotion of tumorigenesis and related phenotypes in vivo include the use of cDNA or short hairpin RNA (shRNA) libraries for gain-of-function or loss-of-function screens, respectively. In addition, the CRISPR/Cas9 system, a novel powerful tool for genome editing [74, 75], can be employed for gain-of-function and loss-of-function screens. The conventional CRISPR/Cas9 system uses a short guide RNA (sgRNA) to direct the Cas9 DNA endonuclease to a complementary DNA target, resulting in double-strand DNA cleavage, which can result in loss-of-function frameshift indels within exons when DNA breaks are repaired by error-prone non-homologous end-joining mechanisms. Alternative Cas9 enzymes, lacking endonuclease activity, have been engineered that promote transcriptional repression [76, 77] or activation [78–80] of target genes when coexpressed with targeting sgRNAs. These approaches have several advantages and disadvantages compared with TMIM, and the different approaches thus provide complementary technologies for cancer gene discovery (Table 2).

**Table 2:** Comparison of genome-wide TMIM, CRISPR/Cas9 and shRNA/cDNA expression technologies

Feature	TMIM	CRISPR/Cas9	shRNA and cDNA libraries
Screen set-up	Two-component system (transposase and transposon)	Comprehensive delivery of libraries can be technically challenging	Comprehensive delivery of libraries can be technically challenging
Possible types of mutation	Activating and disruptive mutations possible owing to transposon insertion or remobilization	Disruptive mutations (knockout libraries)	Knockdown or overexpression — potentially reversible
		Chromosomal deletions and translocations are possible (knockout libraries)	
	Chromosomal deletions and rearrangements are rare		
		Mutations either can (transcription repression [76, 77] and activation [79, 80] libraries) or cannot be reversed (knockout libraries)	
	Mutations can be reversed following transposon remobilization		
Mutagenesis efficiency	Biallelic gene inactivation rare in diploid cells	Biallelic mutation achievable with knockout libraries [88] and 90–99 % knockdown efficiency achievable with repression libraries [89]	≥70 % gene knockdown with validated shRNA clones [82–84]
			>2 standard deviation overexpression by 90 % of cDNA expression vectors [90]
Undesired and off-target effects	Local hopping effects [31], passenger insertions	Minimal off-target effects [78, 81, 88, 89, 95, 96]	Off-target effects can be significant [97–99]
		Viral-associated insertional mutagenesis possible [93, 94]	Viral-associated insertional mutagenesis possible [93, 94]
Genome coverage	Whole genome in principle, but affected by integration-site preferences, local hopping and chromatin accessibility	Dictated by library design	Dictated by library design
		***Knockout libraries***	~8000 human, ~15,000 mouse genes (NKI shRNA library) [85]; >20,000 human and mouse genes (TRC shRNA library) [82, 83]; ~60,000 human and mouse genes (Hannon–Elledge shRNA library) [84]; >17,000 human genes (cDNA expression library) [90]
		**GeCKOv2** [86]: ~20,000 genes, 1000–2000 miRNAs for human and mouse. **Koike-Yusa** ***et al*** **.** [87]: ~20,000 mouse genes. **Wang** ***et al*** **.** [88]: ~7000 human genes	
		***CRISPR-activation libraries***	
		**CRISPRa** [89]: ~16,000 human genes. **SAM** [78]: all human RefSeq genes	
		***CRISPR-inhibition library*** [89]	
		~16,000 human genes	

*NKI* Netherlands Cancer Institute, *shRNA* short hairpin RNA, *TMIM* transposon-mediated insertional mutagenesis, *TRC* The RNAi Consortium

One major pitfall of shRNA, cDNA and CRISPR/Cas9 screens is that these approaches allow for identification of either tumor suppressors or oncogenes, but not both at the same time [78, 81]. By contrast, TMIM has the ability to detect both tumor suppressors and oncogenes simultaneously owing to the genetic elements within the transposons that intercept and promote transcription (see discussion above). Comprehensive shRNA [82–85], sgRNA [78, 86–89] and cDNA [90] libraries have been created for forward-genetic screens. However, the task of delivery of these libraries to the cell type of interest for in vivo screens is not trivial. Usually, libraries are delivered in vitro, followed by orthotopic or subcutaneous transplantation of the library-infected cells [91]. While this can be a viable approach in many cases, it might not always accurately recapitulate tumor progression in its natural environment [92] and might therefore select for false-positive candidate cancer genes. In addition, delivery of libraries with lentiviruses can cause tumor-promoting insertional mutagenesis [93, 94] that remains undetected unless these insertion sites are mapped in conjunction with shRNA/sgRNA/cDNA identification. TMIM does not face the issue of library delivery as the transposons are already included in the genome of transgenic mouse strains, and transposon mobilization is readily achieved in virtually any cell type. However, owing to the local hopping effect [34] observed in TMIM, the donor chromosome containing the parental transposon concatemer has to be excluded from the analysis. Thus, to probe all chromosomes by TMIM, more than one transposon mouse strain has to be used [31].

Another bias of shRNA and CRISPR/Cas9 screens is that shRNAs and sgRNAs are designed to target specific sequences. Thus, these screens are inherently biased, although whether this impacts candidate cancer gene discovery remains to be determined. Moreover, while shRNA and sgRNA design algorithms generate sequences with minimal predicted off-target effects, such effects cannot be excluded experimentally [78, 81, 88, 89, 95–99]. To control for off-target effects by shRNA and sgRNAs, bona fide hits need to be identified by more than one shRNA or sgRNA. In TMIM, the number of transposon insertion sites in a predefined genomic window determines the statistical significance of a CIS [39–41, 43]. However, owing to the continued hopping of unselected transposons and the consequential heterogeneity of tumors with hundreds of passenger insertions, accurate CIS calling remains challenging. Not only are bona fide candidate cancer genes excluded and false positives included following the statistical analysis, CISs might also affect more than one gene. Thus, proper functional validation of any candidates identified by these screening methods is an absolute requirement.

Current sgRNA, shRNA and cDNA libraries are fairly comprehensive, but they do not yet match the ability of TMIM to query virtually the entire genome. However, it is difficult to identify small genetic entities such as microRNAs and enhancers because the likelihood of transposon insertions in the precise locations that would affect their expression or activity is lower. With the CRISPR/Cas9 system, these genes and genetic elements can be targeted and inactivated directly. Indeed, commercially available CRISPR/Cas9 libraries already contain sgRNAs targeting microRNAs [86], and libraries targeting other genetic elements will surely be developed in the near future. Another consideration is that complete target repression is not achieved by either shRNA or TMIM. shRNAs vary drastically in their ability to repress target mRNAs, and transposon insertion is typically observed in only one allele. These technologies are thus biased towards the identification of candidate cancer genes whose incomplete repression promotes tumorigenesis, such as haploinsufficient tumor suppressors or tumor suppressors that readily undergo loss-of-heterozygosity. Conversely, the CRISPR/Cas9 system readily generates biallelic deletions [88, 100] and is therefore able to discover genes that will yield phenotypes only after homozygous loss. Thus, genome coverage and gene dosage are important considerations when choosing a screening system.

Finally, insertions and deletions introduced by the CRISPR/Cas9 system occur through error-prone non-homologous end joining [75]. It is therefore possible that in-frame indels are generated that do not abrogate protein expression [87] but alter proper biological function. This, in turn, could yield different phenotypes compared with those arising from the absence of the protein and could affect the outcome and/or interpretation of the screen. In-frame indels will be selected for if they provide a biological advantage, and are therefore distinguishable from indels that result in frameshifts. Such in-frame indels might reveal interesting aspects about the biology of certain proteins; however, their relevance to human disease will have to be determined on a case-by-case basis. In summary, the different technologies for forward-genetic screening have various pros and cons that need to be considered when designing a screen experiment.

## Concluding remarks

The past few years have brought remarkable advances in the field of transposon-mediated insertional mutagenesis. First, technological developments have enabled investigators to identify cancer genes in an ever-expanding array of cell types and at various stages of tumor evolution. Second, improvements to bioinformatics and statistical methods ensure the identification of crucial cancer genes and pathways and the exclusion of false-positive hits. Third, the vastly increased availability of genomic and mutational data from human cancer specimens allows for the comparison of such data with TMIM results, thereby distinguishing genes with relevance to human cancer that were identified in mice. TMIM remains relevant in the age of CRISPR/Cas9 screens, and together these technologies form a powerful and complementary toolbox to query the genome for the genetic causes of cancer.

## Abbreviations

CIS: Common insertion site
CRC: Colorectal cancer
CRISPR/Cas9: Clustered regularly interspaced short palindromic repeats/CRISPR-associated protein 9
ES: Embryonic stem
gCIS: Gene-centric common insertion site
MAPK: Mitogen-activated protein kinase
MSCV: Murine stem cell virus
pA: Polyadenylation signal
PB: *piggyBac*
PGK: Phosphoglycerate kinase 1
PI3K: Phosphoinositide 3-kinase
PRIM: Poisson regression insertion model
QIseq: Quantitative insertion site sequencing
SA: Splice acceptor
SB: *Sleeping Beauty*
SD: Splice donor
sgRNA: Short guide RNA
shRNA: Short hairpin RNA
TSS: Transcriptional start site
